# Bland Embolization of Benign Liver Tumors: Review of the Literature and a Single Center Experience

**DOI:** 10.3390/jcm10040658

**Published:** 2021-02-09

**Authors:** Daniel Crawford, Sailen Naidu, Indravadan Patel, Grace Knuttinen, Sadeer Alzubaidi, Rahmi Oklu

**Affiliations:** 1 Division of Vascular and Interventional Radiology, Mallinckrodt Institute of Radiology, Washington University in St. Louis, St. Louis, MO 63110, USA; daniel.crawford@wustl.edu; 2 Division of Vascular and Interventional Radiology, Mayo Clinic Arizona, Phoenix, AZ 85054, USA; Patel.Indravadan@mayo.edu (I.P.); knuttinen.grace@mayo.edu (G.K.); Alzubaidi.Sadeer@mayo.edu (S.A.); oklu.rahmi@mayo.edu (R.O.)

**Keywords:** vascular and interventional radiology, embolization, neoplasms, adenoma, focal nodular hyperplasia, hemangioma, liver

## Abstract

Transarterial embolization has shown promise as a safe, effective, and less invasive treatment modality for benign liver lesions (hemangioma, focal nodular hyperplasia (FNH), and hepatic adenoma (HA)) with fewer complications compared to surgical intervention. There is no consensus regarding the most appropriate embolization material(s) for the treatment of benign liver tumors. The purpose of this study was to review the current literature regarding the transarterial embolization of benign liver tumors and to share our single center experience. This was a non-blinded, retrospective, single-institution review of the bland embolization of benign liver tumors. Clinical data and imaging before and after embolization were used to evaluate lesion response to transarterial embolization. Twelve patients were included in the study. Five patients with six hemangiomas were treated. Pain was a presenting complaint in all five of these patients. The median change in tumor volume was −12.4% and ranged from −30.1% to +42.3%. One patient with two FNH lesions was treated, and both lesion volumes decreased by more than 50%. Six patients with 10 adenomas were treated. Pain was a presenting complaint in three patients, and five patients had a lesion >5 cm. The median change in tumor volume was −67.0% and ranged from −92.9% to +65.8%. Bland transarterial embolization of liver hemangiomas, FNH, and HA can be an effective and minimally invasive treatment modality to control the size and/or symptoms of these lesions. There is a variable response depending on tumor type and the embolization materials used.

## 1. Introduction

Benign liver tumors are relatively common. Most are found incidentally during imaging for other indications. Hemangioma, focal nodular hyperplasia (FNH), and hepatic adenoma (HA) make up the majority of the benign liver lesions. Each differs in cellular composition and indications for intervention.

Hemangioma represents the most common benign liver lesion with a prevalence estimated between 0.4–20% [1]. The vast majority of hemangiomas are found incidentally during imaging for other indications and do not require follow up imaging or treatment. Rare indications for treatment include Kasabach-Merritt syndrome (consumptive thrombocytopenia) or bulk related symptoms such as pain, fullness, shortness of breath, and early satiety. Hemangiomas are fed by hepatic arteries and contain numerous vascular spaces lined with flattened epithelium. On contrast-enhanced cross-sectional imaging or contrast-enhanced ultrasound, hemangiomas demonstrate peripheral discontinuous enhancement with progressive fill-in over time. On magnetic resonance imaging, they are hyperintense on T2-weighted sequences. Giant hemangiomas (greater than 10 cm in diameter) may contain extensive fibrous changes as well as calcifications [2,3].

Focal nodular hyperplasia is the second most common benign liver lesion with a prevalence of 0.4–3.0% [1]. FNH has a significant female preponderance (up to 90%) and is thought to represent a polyclonal cellular response to a dystrophic artery. The majority of FNH are stable in size, rarely symptomatic, and do not have malignant potential. Indications for therapy are rare but include symptoms such as pain or fullness attributable to the FNH. On magnetic resonance imaging, FNH lesions have a lobulated border, are generally isointense on T2- and T1-weighted non-contrast imaging, show hyperenhancement on the arterial phase, and are relatively isointense to the liver on portal venous phase and delayed imaging. In about half of all FNH lesions, a central scar is identified which can demonstrate delayed enhancement due to fibrous tissue [2,3].

Hepatic adenoma (HA) is the least common benign liver tumor with a prevalence of 0.001–0.004%. They frequently occur in young women with a history of prolonged oral contraceptive use [4]. While classified as benign, HA have a known propensity to bleed, particularly when over 5 cm in size [1]. In addition, HA may undergo malignant transformation. Given these factors, adenomas require intervention more frequently, particularly when they are above 5 cm in diameter. Several subtypes of HA have been identified based on genetic mutations and portend different risks of hemorrhage and malignant transformation. These subtypes include inflammatory (I-HCA), HNF-1α (H-HCA), and β-catenin activated HCA (β-HCA) [1,4]. The imaging characteristics vary according to the subtype. Magnetic resonance imaging is often the best imaging modality for diagnostic purposes due to the ability to assess the signal intensity of T1- and T2-weighted imaging, the presence of fat, hemorrhage or necrosis, and the enhancement pattern on multiphase imaging [2,3].

Historically, surgical resection of benign liver tumors was the only available treatment option, but it came with a high complication rate of 9–25% [5,6]. Transarterial embolization of benign liver tumors was first described in 1979 [7] and has shown promise as a safe, effective, and less invasive treatment modality with fewer complications compared to surgical intervention. Multiple studies have shown tumor regression and symptomatic improvement with this treatment, thereby foregoing the need for surgical intervention [8].

There is no consensus regarding the most appropriate embolization material(s) for the treatment of benign liver tumors [9]. A wide variety of embolization materials have been reported in the literature to treat different benign tumors. For hemangiomas, particulate embolics including polyvinyl alcohol (PVA), trisacyl gelatin microspheres, gel foam, and coils have been described. More recently, lipiodol and bleomycin have been utilized with good results. [8,9,10,11]. In FNH and adenoma, particulate embolic materials with or without lipiodol have been well described [8,9]. In order to improve the transarterial treatment of benign liver tumors, there is a continued need to identify the most appropriate material(s) to use for embolization.

The purpose of this study was to review the current literature regarding the embolization of benign liver tumors and to share our single center experience with bland embolization of benign liver tumors. Measured outcomes include changes in tumor size and symptomatic improvement after bland transarterial embolization.

## 2. Material and Methods

Institutional review board approval was received for this study. This was a retrospective, single-institution review of bland embolization of benign liver tumors (hemangiomas, FNH and HA). Any patient that underwent non-emergent, non-bleeding, bland transarterial embolization of a benign liver tumor from September 2009 through October 2018 was included in the study. Patients who underwent multiple tumor embolizations were included. Patient demographics are reported in Table 1.

The femoral artery was accessed using the Seldinger technique. Angiography of the superior mesenteric and celiac arteries was performed to identify the arterial anatomy and vessels contributing blood flow to the tumor. Super-selective catheterization was used to access the feeding vessel and the tumor was embolized until flow stasis was observed. This was repeated for other feeding vessels and/or tumors if applicable. Two to three treatment sessions were required in several patients due to the size of the liver lesion (Table 1). Embolization was performed using several materials, including polyvinyl alcohol (PVA) particles (Cook, Bloomington, IN and Contour—Boston Scientific Corporation, Marlborough, MA, USA), tris-acryl gelatin microspheres (Embosphere, Merit Medical Systems, Inc., South Jordan, UT, USA), and ethiodol (Guerbert, LLC, Villepinte, France).

Procedural details including post-procedure complications were recorded. The electronic medical record was reviewed to assess for symptomatic relief when applicable. Cross-sectional imaging before the embolization(s) and all subsequent imaging studies after the embolization were reviewed. Tumor size was measured as the greatest axial diameter and the corresponding greatest perpendicular distances in the axial and craniocaudal dimensions. The measurements included any enhancing tissue, as well as the devascularized core of the lesion. Percent change in lesion volume was calculated in reference to the baseline lesion size:

∆V = ((d_1_d_2_d_3_)_b_ − (d_1_d_2_d_3_)_f_)/(d_1_d_2_d_3_)_b_ × 100

where ∆V equals change in lesion volume, b is baseline, f is final, and d_1_, d_2_, and d_3_ are the three orthogonal lesion dimensions. Response evaluation criteria in solid tumors (RECIST) were also used to evaluate lesion response using the greatest measured distance [12]. Data were analyzed using standard statistical techniques, including medians/ranges for continuous data and counts/percentages for categorical data.

The five levels of the Society of Interventional Radiology (SIR) Adverse Event Severity Scale [13] were used to classify complications. Mild adverse events (level 1) were classified as requiring no therapy or nominal (non-substantial) therapy, or a near miss. Moderate adverse events (level 2) required moderate escalation of care, requiring substantial treatment. Severe adverse events (level 3) required marked escalation of care (i.e., hospital admission or prolongation of existing hospital admission for >24 h). Life threatening of disabling adverse events (level 4) include cardiopulmonary arrest, shock, organ failure, unanticipated dialysis, paralysis, or loss of limb/organ. Level 5 adverse events involve patient death.

## 3. Results

**Figure 1:**
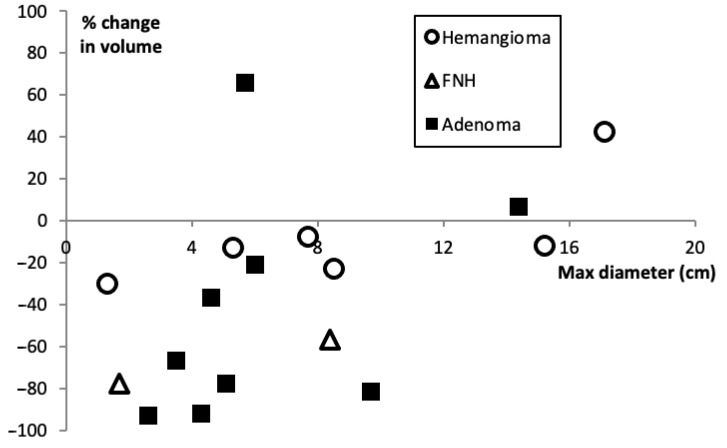
Percent change in lesion volume versus maximum initial diameter for bland transarterial embolization of hepatic hemangiomas, FNH, and adenomas. A negative percent change indicates that the lesion decreased in volume.

A total of 12 patients were included in the study. There were five patients with hemangiomas, one with FNH, and six patients with adenomas (Table 1). Follow-up ranged from 0–69 months and the change in tumor volume ranged from −92.9% to +65.8%. The percent change in tumor volume versus maximum initial diameter is shown in Figure 1.

### 3.1. Hemangiomas

Five patients with six lesions were treated. Follow-up ranged from 0–45 months. Pain was a presenting complaint in all five patients. Every patient was treated with PVA particles and one patient also received ethiodol. The median change in tumor volume was −12.4% and ranged from −30.1% to +42.3%. As per RECIST, all lesions were stable. One patient had asymptomatic bradycardia overnight after embolization, which was most likely unrelated to the procedure (SIR Adverse Event Severity Scale level 1). There were no other complications. Two patients were lost to follow-up, one patient had persistent right upper quadrant pain, and one patient has plans for surgical resection.

### 3.2. Focal Nodular Hyperplasia

**Figure 2:**
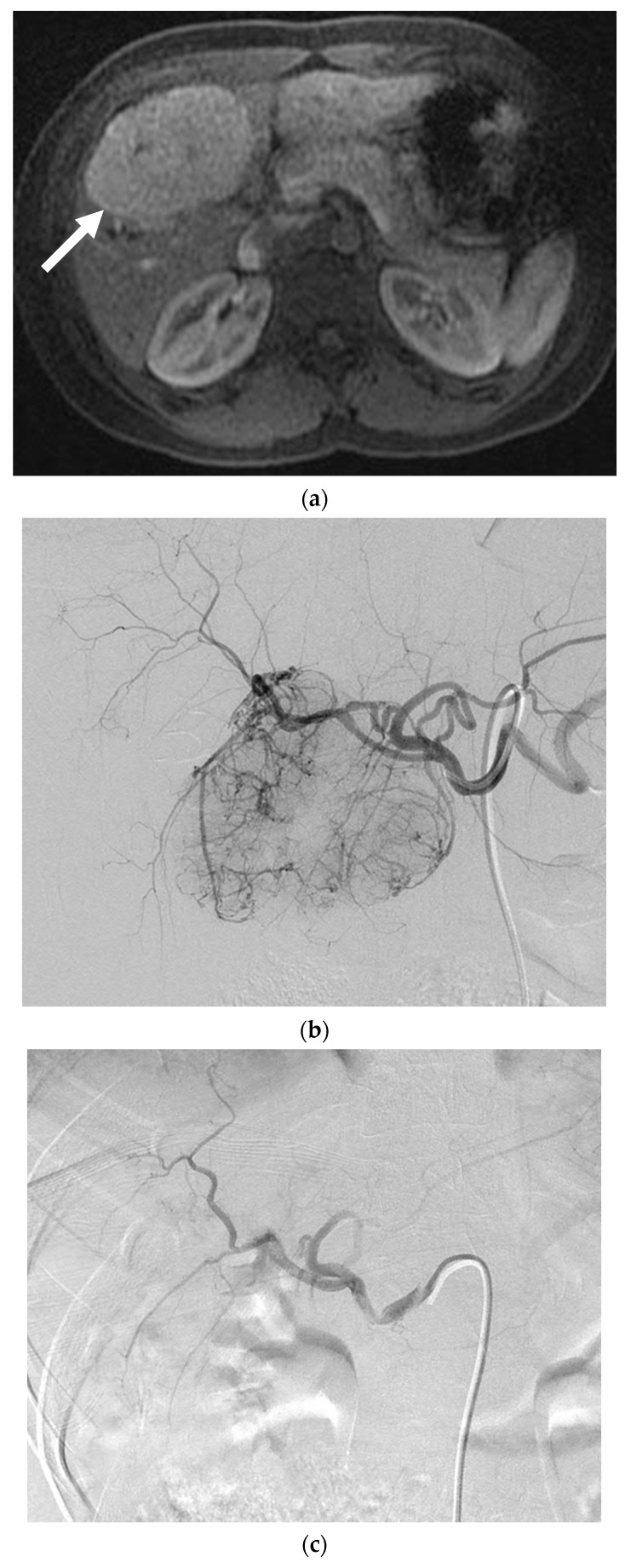

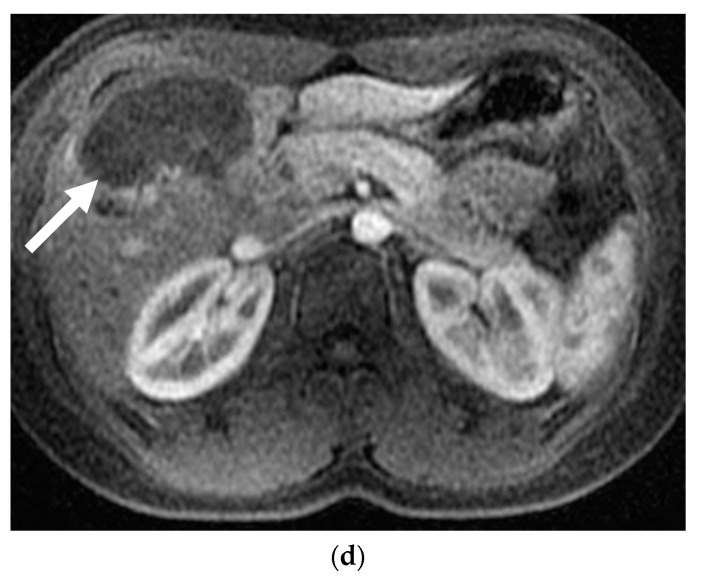
A 24-year-old female with focal nodular hyperplasia (FNH) and right upper quadrant pain. (**a**) Magnetic resonance imaging (MRI) with contrast demonstrates a hypervascular lesion in segments 4 and 5 (solid white arrow). Twenty-minute delayed images showed continued enhancement (not shown). (**b**) Angiogram reveals segment 4 and segment 5 arteries that feed a hypervascular mass. Each artery was infused with 200 and 300 µm PVA particles until stasis. (**c**) Post-embolization angiogram shows no flow to the FNH. (**d**) MRI with contrast one month after embolization reveals no residual enhancement and a decrease in the size of the FNH (solid white arrow). The patient’s pain resolved following the embolization.

One patient with two lesions was treated with PVA particles for the treatment of abdominal pain (Figure 2). Both lesion volumes decreased by more than 50%. As per RECIST, one lesion was stable and one had a partial response. The patient had resolution of her abdominal pain at her one-month follow-up. There were no complications.

### 3.3. Adenomas

**Figure 3:**
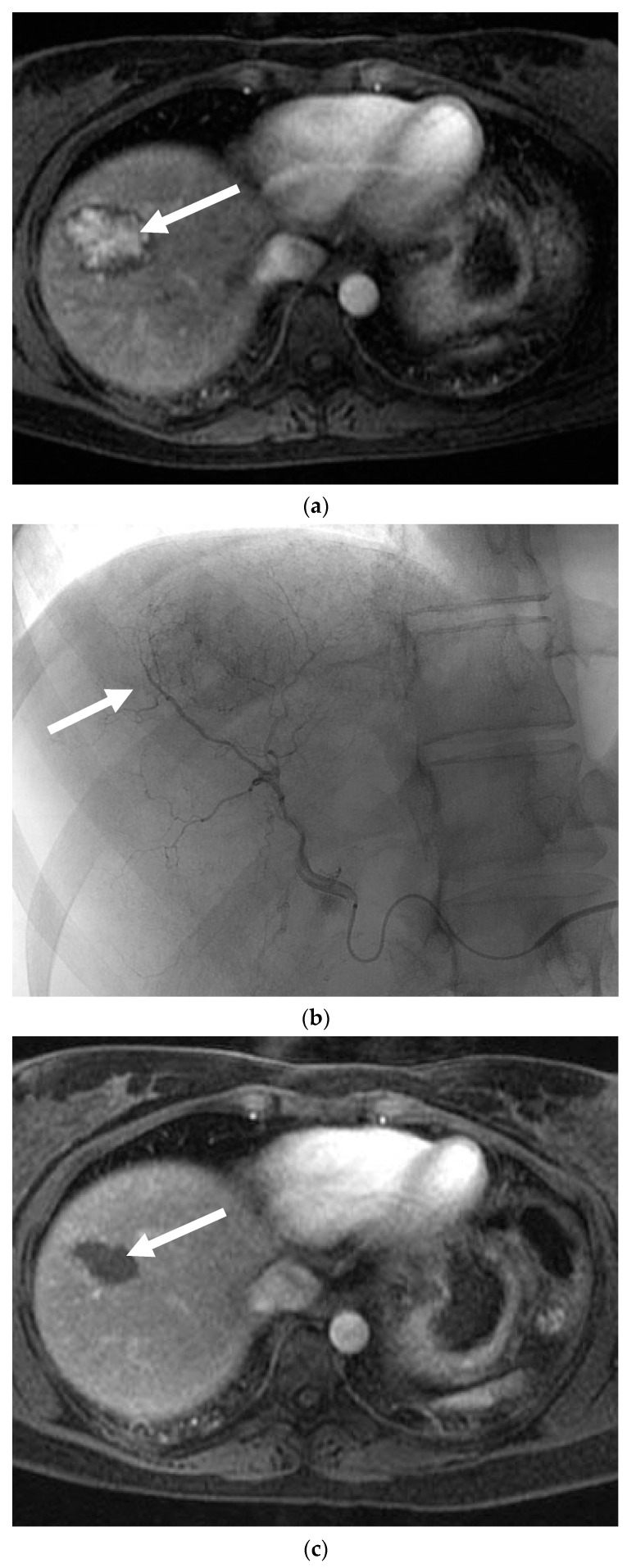
Hepatic adenoma following embolization. (**a**) MRI with gadolinium shows an enhancing 4.3 cm lesion in segment 8 (solid white arrow). This was symptomatic and had increased in size over the past year. (**b**) Selective injection of the right hepatic artery demonstrating the hypervascular hepatic adenoma (solid white arrow). This was treated with 40–100 µm and 100–300 µm tris-acryl gelatin microspheres. (**c**) Three months following embolization, MRI with contrast shows complete necrosis of the adenoma with interval decrease in size (solid white arrow).

Six patients with 10 lesions were treated; imaging from one case is shown in Figure 3. Follow-up ranged from 0–69 months. Pain was a presenting complaint in three patients and five patients had a lesion greater than 5 cm in diameter. Two patients reported oral contraceptive use for greater than six months. Four of the six patients were treated with Embospheres and two were treated with PVA particles +/− ethiodol. The median change in tumor volume was −67.0% and ranged from −92.9% to +65.8%. The median volume changes for spherical particles (five lesions) and PVA (four lesions) were −77.9% and −15.1% respectively. As per RECIST, three lesions were stable, five had a partial response, and one had progressive disease. There was one confirmed complication of postembolization syndrome and one possible case of *Staphylococcus* bacteremia that was treated at an out of state hospital (SIR Adverse Event Severity Scale levels 1 and 4, respectively [13]). One patient was lost to follow-up and one patient had a subcapsular bleed in the region of the treated adenoma 49 months after the index procedure; it is uncertain if this represented a delayed bleed from the treated tumor or from a separate adenoma in the same region.

## 4. Discussion

The bland transarterial embolization of benign liver tumors has variable effectiveness at reducing tumor size. Tumor response depends on tumor type and likely the embolic agent utilized. In this study, three different types of benign tumors were treated with PVA particles or microspheres and resulted in tumor volume changes ranging from −92.9% up to +65.8%. There were minimal complications with bland embolization and one case of postembolization syndrome.

### 4.1. Hemangiomas

The European Association for the Study of the Liver (EASL) clinical guidelines [1] report that there is no relationship between the size of hemangiomas and complications; thus, the only indications for treatment of liver hemangioma are Kasabach-Merrit syndrome, growing lesions, or symptomatic lesions compressing adjacent structures. All five patients in this study had symptomatic lesions greater than 5 cm in diameter. Two patients experienced pain relief, one had persistent right upper quadrant pain, and two patients were lost to follow-up. One patient had pre-existing anemia that resolved after embolization.

The largest hemangioma in this study (17.1 cm) grew after two interventions. This large tumor had an abundance of feeding vessels from the left hepatic artery, right hepatic artery, and branches off the superior mesenteric artery and common hepatic artery. More than five of these branches were embolized with PVA particles during the treatments, but there were still multiple feeding vessels that were too small to cannulate for embolization. The persistent growth was likely due to the known incomplete embolization.

Despite the small modest reduction in hemangioma volume observed in this study, bland embolization was effective at reducing or eliminating pain in two patients. The median volume reduction of the six lesions was 12.4%. This was less than that reported in other studies using bleomycin and lipiodol, where a median volume reduction of 51% in 25 patients was reported [11]. In another study, a greater than 50% reduction in all 29 treated lesions was noted [10]. Li et al. [14] report an average diameter decrease of 62.5% in 1120 lesions using a mixture of lipiodol and pingyangmycin (a bleomycin-like drug not available in the United States). The improved response may be due to the sclerosing effects of bleomycin and the retention effect of lipiodol [10]. This combination may be a better choice for transarterial embolization of liver hemangiomas. However, while sclerosants may be more effective for hemangiomas due to the theoretical benefit of forming intraluminal thrombi to occlude the lesion [15], this comes with the increased potential risks of sclerosing cholangitis, interstitial pneumonia, and/or pulmonary fibrosis [9].

Given the results of the previous studies showing the significant size reduction with bleomycin and lipiodol embolization, use of a sclerosant appears to be a superior regimen for hemangiomas as compared to bland particulate embolization.

### 4.2. Focal Nodular Hyperplasia

The EASL guidelines note that patients with a firm diagnosis of asymptomatic FNH do not need routine follow up [1]. If patients are symptomatic, there is evidence of obstructed vessels or biliary ducts, or if there is uncertainty in the diagnosis, they recommend referral to a liver tumor multidisciplinary team for evaluation [16]. In the current study, only one patient with two FNH lesions presented for transarterial bland embolization due to persistent right upper quadrant pain. Both lesions responded well to bland embolization with a decrease in volume by more than 50%. Additionally, this patient had significant pain relief. Birn et al. [17] performed bland embolization of 17 FNH lesions with microspheres and report a 61% mean decrease in lesion size after 4–10 weeks. Vogl et al. [18] reported complete FNH resolution in two out of four patients, a 67% decrease in one, and no change in another with PVA embolization. Zhang et al. [15] used bleomycin-lipiodol with PVA particles and reported an average diameter decrease of 36% in 27 patients and significant symptomatic improvement in all patients. The role for bleomycin in FNH is less certain than in hemangiomas, and the increased risk of side effects may outweigh its potential benefits. As such, based on our limited experience and the experience from other centers, bland embolization may be adequate for significant size reduction and symptomatic relief in FNH.

### 4.3. Adenomas

The EASL guidelines [1] recommend that hepatocellular adenoma be treated when 5 cm or greater in females due to the risk of malignant transformation or rupture, and that any adenoma in males be treated due to its increased risk of malignant transformation. In our series, two males were treated with embolization as both were felt to be poor surgical candidates. All but one patient in this study had an adenoma greater than 5 cm; the indication for treatment in this patient was pain. Symptoms resolved after two treatments. Seven of nine lesions (78%) decreased in size after embolization, with a median volume decrease of 67%. Other studies have reported size decreases ranging from 30% to 90% [8], and none reported tumor growth. It is uncertain why there were two tumors in the present study that grew after treatment. One growing lesion was very large (14.4 cm maximum dimension) and had several tortuous feeding vessels, which made the embolization more complex. The other non-responsive lesion grew despite two interventions, including one with ethiodol that demonstrated stasis within the tumor. This patient eventually presented for surgical resection and was found to have no evidence of malignant transformation. Several other lesions in this patient with hepatic adenomatosis responded appropriately to transarterial embolization.

The variable response for adenoma following embolization may be multifactorial. While continued growth certainly could be due to inadequate embolization, adenomas do differ at the molecular and genetic level, impacting their behavior. The inflammatory subtype (I-HCA) accounts for 40–55% of all adenomas and has the highest risk of hemorrhage. HCA inactivated for HNF-1α (H-HCA) represents 30–40% of adenomas and is the least aggressive subtype with a low risk of complications. The β-catenin activated HCA subtype (β-HCA) has the highest risk of malignancy [1,4]. Accordingly, the risk of malignant degeneration should factor into the treatment decision for this adenoma subtype; the risk-benefit profile for a given lesion may favor curative measures such as surgical resection or radiofrequency ablation rather than bland embolization. It is possible that some subtypes may respond better to embolization relative to others; however, this study was not powered to answer this question.

The median diameter of the adenomas treated in this series is larger than other reported series in the literature. For example, in one retrospective study of 100 adenomas treated with embolization or resection, 70 were less than 5 cm in size, and the median diameter in the embolization group was 2.6 cm [19]. The current recommendation is to treat HA when larger than 5 cm in diameter due to the increased risk of hemorrhage and malignant transformation. Certain scenarios warrant intervention at smaller diameters, such as multiple adenomas, the need for continued hormone therapy, recurrence following surgery, or symptomatic patients. This has relevance in the treatment of HA because ablation is a viable treatment option when the lesion is less than 4 cm in diameter. A number of studies have demonstrated very good tumor control with the use of microwave or radiofrequency ablation [20,21,22]. The median diameters of hepatic adenomas in these studies were 2.1 cm [20], 2.7 cm [21], and 3.0 cm [22]. In our institution, there were few adenomas smaller than 3.0 cm that underwent treatment with embolization. Most patients with such lesions underwent conservative management, including withdrawal of any offending agent and surveillance imaging.

In our experience, each adenoma treated with spherical particles decreased in size, whereas several adenomas treated with PVA did not decrease in size. In a study by Deodhar et al. [23], smaller spherical particles (40–120 um) were utilized in 6/8 patients in 17 embolization sessions, with the regression of 13/16 treated adenomas. The use of small particles may be more effective due increased distal embolization causing ischemia. Similarly, polyvinyl alcohol (PVA) has a tendency to clump, which can lead to a more proximal embolization. PVA was used in a number of the adenoma cases early in our experience and has since been abandoned in favor of spherical particles.

There was one case of bleeding following embolization of an adenoma. This lesion was embolized using bland particles and the lesion decreased substantially in size. However, approximately four years after the embolization, the patient presented with a subcapsular bleed that was presumably due to the adenoma despite its small size (2 cm × 3 cm). While it is assumed that the risk of bleeding is decreased with the decreased size of a lesion, there may be some continued risk of bleeding despite embolization [8]. No study has been able to demonstrate a reduction in malignant transformation in patients treated with bland embolization.

The several limitations of this study include its retrospective nature, the small sample size, lack of follow-up in several patients, and variability in the type and size of the embolization particles utilized. Management of benign liver tumors should follow established clinical guidelines, and the option of performing transarterial embolization should not circumvent recommendations regarding when to intervene in these lesions. The decision to perform transarterial embolization rather than surgical resection for this study often came from the perceived increased risk of surgery due to patient body habitus or proximity to adjacent structures. Future beneficial work could include a comparison between the surgical and transarterial treatment of benign liver lesions, specifically adenomas and hemangiomas.

## 5. Conclusions

Bland transarterial embolization of liver hemangiomas, FNH and adenomas can be an effective and minimally invasive treatment modality to control the size and/or symptoms of these lesions. Hemangiomas undergo a modest size decrease in tumor size with symptomatic improvement using bland embolization; however, the addition of a sclerosing agent may be beneficial. Symptomatic FNH lesions responded well both dimensionally and symptomatically to bland embolization. Adenomas have a variable response to bland embolization, but super-selective bland embolization with smaller spherical particles can be effective in certain scenarios and delay or obviate the need for surgery.

## Figures and Tables

**Table 1:** Demographic data, indications for transarterial embolization, procedural details, lesion size and change in volume, and outcomes for bland transarterial embolization of hepatic hemangiomas, focal nodular hyperplasia (FNH), and hepatic adenomas. “S” refers to liver segment when applicable. The adenoma tumor subtype is listed in parentheses for lesions with known pathology. I-HCA = inflammatory subtype, H-HCA = HNF-1α subtype, PVA: polyvinyl alcohol, RUQ: right upper quadrant.

Tumor Type	Age, Sex	Indication	Treat-ment Sessions	Embolization Particles	Initial Lesion Dimensions, cm (Location)	% Change in Lesion Volume	Follow-Up (Months) Imaging, Clinical	Clinical Outcome
Hem-angioma	43, F	Back pain; lesion growth	2	PVA: 200 µm	15.9 × 12.2 × 17.1 (S4)	+42.3%	38, 45	Pain improved; plans for surgical resection given size of hemangioma
Hem-angioma	64, F	RUQ pain; anxiety regarding rupture	2	PVA: 300–500 µm, followed by 500–700 µm	15.2 × 10.5 × 12.5 (entire R lobe)	−11.9%	7, 0	Lost to clinical follow up
Hem-angioma	55, F	RUQ pain	1	PVA: 200 µm	6.7 × 6.0 × 7.7 (S6)	−7.6%	19, 19	Persistent RUQ pain
Hem-angioma	52, F	Abdominal pain; anemia	1	PVA: 100 µm	8.5 × 6.0 × 8.1 (S4)	−22.7%	12, 11	Decreased abdominal pain; resolution of anemia
Hem-angioma	46, F	Abdominal pain and fullness	1	PVA: 200 µm Ethiodol	5.2 × 4.8 × 5.3 (S6) 1.3 × 1.1 × 1.1 (S7)	−12.9% (S6) −30.1% (S7)	16, 0	Lost to clinical follow up
FNH	24, F	RUQ pain	1	PVA: 200 µm, 300 µm	8.4 × 5.4 × 7.2 (S8) 1.6 × 1.2 × 1.7 (S5)	−57.0% (S8) −77.9% (S5)	1, 1	Pain resolved
Adenoma (I-HCA)	20, M	Adenoma > 5 cm; adenomatosis	1	Embospheres: 40–120 µm followed by 100–300 µm	3.5 × 2.2 × 2.7 (S4) 6.0 × 4.5 × 5.4 (S1)	−67.0% (S4) −21.2% (S1)	9, 15	Pain resolved
Adenoma (I-HCA)	32, F	Pain; lesions > 5 cm, growing	2	PVA: 200 µm Ethiodol (1st procedure only)	12.9 × 9.4 × 14.4 (S7) 9.7 × 7.1 × 7.7 (S6)	+6.7% (S7) −81.6% (S6)	3, 6	Segmental resection (S7) due to enlarging adenoma
Adenoma (unknown subtype)	30, F	Pain	2	Embospheres: 40–120 µm followed by 100–300 µm	4.3 × 3.1 × 3.3 (S8) 2.6 × 1.8 × 1.9 (S4)	−92.3% (S8)/ −92.9% (S4)	21/18, 18	Pain resolved; recurrent 1.8 cm lesion in S8?
Adenoma (H-HCA)	22, F	Adenoma > 5 cm; adenomatosis	1	Embospheres: 100–300 µm, followed by 300–500 µm	5.8 × 4.5 × 5.9 (S6)	Unknown	N/A, 0	All clinical and imaging follow up performed at out of state hospital
Adenoma (unknown subtype)	39, F	Adenoma > 5 cm	1	Embospheres: 100–300 µm	5.1 × 4.1 × 3.3 (S7)	−77.9%	49, 69	Subcapsular hematoma after 49 months originating from embolized adenoma, treated conservatively
Adenoma (I-HCA)	30, M	Acute abdominal pain; drop in hemoglobin	3	PVA: 200 µm (procedure 1); PVA: 200 µm + ethiodol (procedure 2); contour: 150–250 µm + ethiodol (procedure 3)	5.7 × 4.6 × 5.7 (L liver) 4.1 × 2.5 × 4.6 (R liver)	+65.8% (L liver)/ −36.8% (R liver)	12/4, 17	Left hepatectomy due to increased adenoma size, passed away after status epilepticus during recovery

## Data Availability

The data presented in this study are available on request from the corresponding author.
